# Left frontal hub connectivity delays cognitive impairment in autosomal-dominant and sporadic Alzheimer’s disease

**DOI:** 10.1093/brain/awy008

**Published:** 2018-02-15

**Authors:** Nicolai Franzmeier, Emrah Düzel, Frank Jessen, Katharina Buerger, Johannes Levin, Marco Duering, Martin Dichgans, Christian Haass, Marc Suárez-Calvet, Anne M Fagan, Katrina Paumier, Tammie Benzinger, Colin L Masters, John C Morris, Robert Perneczky, Daniel Janowitz, Cihan Catak, Steffen Wolfsgruber, Michael Wagner, Stefan Teipel, Ingo Kilimann, Alfredo Ramirez, Martin Rossor, Mathias Jucker, Jasmeer Chhatwal, Annika Spottke, Henning Boecker, Frederic Brosseron, Peter Falkai, Klaus Fliessbach, Michael T Heneka, Christoph Laske, Peter Nestor, Oliver Peters, Manuel Fuentes, Felix Menne, Josef Priller, Eike J Spruth, Christiana Franke, Anja Schneider, Barbara Kofler, Christine Westerteicher, Oliver Speck, Jens Wiltfang, Claudia Bartels, Miguel Ángel Araque Caballero, Coraline Metzger, Daniel Bittner, Michael Weiner, Jae-Hong Lee, Stephen Salloway, Adrian Danek, Alison Goate, Peter R Schofield, Randall J Bateman, Michael Ewers

**Affiliations:** 1 Institute for Stroke and Dementia Research, Klinikum der Universität München, Ludwig-Maximilians-Universität LMU, Feodor-Lynen Straße 17, 81377 Munich, Germany; 2 German Center for Neurodegenerative Diseases (DZNE), Magdeburg, Germany; 3 German Center for Neurodegenerative Diseases (DZNE), Bonn, Sigmund-Freud-Str. 27, 53127 Bonn, Germany; 4 Department of Psychiatry, University of Cologne, Medical Faculty, Kerpener Strasse 62, 50924 Cologne, Germany; 5 German Center for Neurodegenerative Diseases (DZNE, Munich), Munich, Germany; 6 Department of Neurology, Ludwig-Maximilians-Universität München, Munich, Germany; 7 Munich Cluster for Systems Neurology (SyNergy), Munich, Germany; 8 Biomedical Center, Biochemistry, Ludwig-Maximilians-Universität München, Munich, Germany; 9 Department of Radiology, Washington University in St Louis, St Louis, Missouri, USA; 10 Knight Alzheimer’s Disease Research Center, Washington University in St. Louis, St. Louis, MO, USA; 11 Hope Center for Neurological Disorders, Washington University in St. Louis, St. Louis, MO, USA; 12 The Florey Institute, The University of Melbourne, Parkville, Victoria, Australia; 13 Department of Psychiatry and Psychotherapy, Ludwig-Maximilians-Universität München, Nußbaumstr. 7, 80336 Munich, Germany; 14 Neuroepidemiology and Ageing Research Unit, School of Public Health, The Imperial College of Science, Technology and Medicine, Exhibition Road, SW7 2AZ London, UK; 15 West London Mental Health Trust, 13 Uxbridge Road, UB1 3EU London, UK; 16 Department of Psychiatry and Psychotherapy, University of Bonn, Sigmund-Freud-Str. 25, 53127 Bonn, Germany; 17 Department of Neurodegeneration and Geriatric Psychiatry, University of Bonn, Sigmund-Freud-Str. 25, 53127 Bonn, Germany; 18 German Center for Neurodegenerative Diseases (DZNE), Rostock, Germany; 19 Department of Psychosomatic, University of Rostock, Gehlsheimer Str. 20, 18147 Rostock, Germany; 20 Institute of Human Genetics, University of Bonn, 53127, Bonn, Germany; 21 Dementia Research Centre, University College London, Queen Square, London, UK; 22 Hertie Institute for Clinical Brain Research, Tübingen, Germany and German Center for Neurodegenerative Diseases (DZNE), Tübingen, Germany; 23 Departments of Neurology, Massachusetts General Hospital, Charlestown HealthCare Center, Charlestown, Massachusetts 02129, USA; 24 Athinoula A. Martinos Center for Biomedical Imaging, Department of Radiology, Massachusetts General Hospital, Charlestown HealthCare Center, Charlestown, Massachusetts 02129, USA; 25 Department of Neurology, University of Bonn, Sigmund-Freud-Str. 25, 53127 Bonn, Germany; 26 Department of Radiology, University of Bonn, Sigmund-Freud-Str. 25, 53127 Bonn, Germany; 27 Section for Dementia Research, Hertie Institute for Clinical Brain Research and Department of Psychiatry and Psychotherapy, University of Tübingen, Tübingen, Germany; 28 Queensland Brain Institute, University of Queensland, Brisbane, Australia; 29 German Center for Neurodegenerative Diseases (DZNE), Berlin, Germany; 30 Department of Psychiatry and Psychotherapy, Charité, Hindenburgdamm 30, 12203 Berlin, Germany; 31 Department of Neuropsychiatry, Charite - Universitätsmedizin Berlin, Charitéplatz 1, 10117 Berlin, Germany; 32 Leibniz Institute for Neurobiology, Magdeburg, Germany; 33 Center for Behavioral Brain Sciences, Magdeburg, Germany; 34 Department of Biomedical Magnetic Resonance, Leipziger Str. 44, 39120 Magdeburg, Germany; 35 German Center for Neurodegenerative Diseases (DZNE), Goettingen, Germany; 36 Department of Psychiatry and Psychotherapy, University Medical Center Goettingen, University of Goettingen, Von-Siebold-Str. 5, 37075 Goettingen, Germany; 37 iBiMED, Medical Sciences Department, University of Aveiro, Aveiro, Portugal; 38 University of California at San Francisco, 505 Parnassus Ave, San Francisco, CA94143, USA; 39 Department of Neurology, University of Ulsan College of Medicine, Asan Medical Center, Seoul, Korea; 40 Department of Neurology, Warren Alpert Medical School of Brown University, Providence, Rhode Island, USA; 41 Department of Genetics and Genomic Sciences, Icahn School of Medicine at Mount Sinai, New York, New York, USA; 42 Ronald M. Loeb Center for Alzheimer’s Disease, Department of Neuroscience, Icahn School of Medicine at Mount Sinai, New York, New York, USA; 43 Neuroscience Research Australia, Barker Street Randwick, Sydney 2031, Australia; 44 School of Medical Sciences, University of New South Wales, Sydney 2052, Australia

**Keywords:** Alzheimer’s disease, cognitive reserve, resting state connectivity, memory, dementia biomarkers

## Abstract

Patients with Alzheimer’s disease vary in their ability to sustain cognitive abilities in the presence of brain pathology. A major open question is which brain mechanisms may support higher reserve capacity, i.e. relatively high cognitive performance at a given level of Alzheimer’s pathology. Higher functional MRI-assessed functional connectivity of a hub in the left frontal cortex is a core candidate brain mechanism underlying reserve as it is associated with education (i.e. a protective factor often associated with higher reserve) and attenuated cognitive impairment in prodromal Alzheimer’s disease. However, no study has yet assessed whether such hub connectivity of the left frontal cortex supports reserve throughout the evolution of pathological brain changes in Alzheimer’s disease, including the presymptomatic stage when cognitive decline is subtle. To address this research gap, we obtained cross-sectional resting state functional MRI in 74 participants with autosomal dominant Alzheimer’s disease, 55 controls from the Dominantly Inherited Alzheimer’s Network and 75 amyloid-positive elderly participants, as well as 41 amyloid-negative cognitively normal elderly subjects from the German Center of Neurodegenerative Diseases multicentre study on biomarkers in sporadic Alzheimer’s disease. For each participant, global left frontal cortex connectivity was computed as the average resting state functional connectivity between the left frontal cortex (seed) and each voxel in the grey matter. As a marker of disease stage, we applied estimated years from symptom onset in autosomal dominantly inherited Alzheimer’s disease and cerebrospinal fluid tau levels in sporadic Alzheimer’s disease cases. In both autosomal dominant and sporadic Alzheimer’s disease patients, higher levels of left frontal cortex connectivity were correlated with greater education. For autosomal dominant Alzheimer’s disease, a significant left frontal cortex connectivity × estimated years of onset interaction was found, indicating slower decline of memory and global cognition at higher levels of connectivity. Similarly, in sporadic amyloid-positive elderly subjects, the effect of tau on cognition was attenuated at higher levels of left frontal cortex connectivity. Polynomial regression analysis showed that the trajectory of cognitive decline was shifted towards a later stage of Alzheimer’s disease in patients with higher levels of left frontal cortex connectivity. Together, our findings suggest that higher resilience against the development of cognitive impairment throughout the early stages of Alzheimer’s disease is at least partially attributable to higher left frontal cortex-hub connectivity.

## Teaser

The neural basis of reserve capacity in Alzheimer’s disease is yet to be fully determined. Franzmeier *et al.* show that greater left frontal hub connectivity within the fronto-parietal control network is associated with greater resilience of cognitive performance during the early stages of autosomal dominant and sporadic Alzheimer’s disease.

## Introduction

Biomarker studies in Alzheimer’s disease have revealed a temporal sequence of the development of brain pathologies and cognitive decline. In autosomal dominantly inherited Alzheimer’s disease, a successive emergence of abnormal CSF concentrations of amyloid-β, tau, microglial activation, grey matter atrophy and cerebral glucose hypometabolism has been reported (Bateman *et al.*, 2012; Benzinger *et al.*, 2013; Suarez-Calvet *et al.*, 2016). Similar findings have been obtained in sporadic late onset Alzheimer’s disease (Jack *et al.*, 2012), providing strong evidence for cascading pathologies that lead to cognitive deficits and ultimately dementia. The extent of cognitive decrease at any level of Alzheimer’s disease pathologies, however, varies considerably between individuals (Vemuri *et al.*, 2011; Mufson *et al.*, 2016). Even in autosomal dominantly inherited Alzheimer’s disease (ADAD), where the course of Alzheimer’s disease development is strongly determined by mutations in genes encoding presinilin-1 (*PSEN1*), presenilin-2 (*PSEN2*) or amyloid precursor protein (*APP*), the cognitive status remains relatively stable in some patients despite advanced levels of amyloid-β and tau pathology (Lim *et al.*, 2016). The theory of reserve posits that such a discrepancy between brain pathology and cognitive impairment is not a prediction error due to incomplete detection of neuronal pathologies, but is due to higher resilience against the effects of pathology (Park and Reuter-Lorenz, 2009; Stern, 2012). In other words, some people may show higher ability to maintain cognitive abilities relatively well at any given level of brain pathology. At the epidemiological level, more years of education, higher general cognitive ability, occupational attainment and other cognitively challenging life experiences (Valenzuela and Sachdev, 2006), or higher physical fitness (Okonkwo *et al.*, 2014; Tolppanen *et al.*, 2015; Duzel *et al.*, 2016) have been associated with higher cognitive abilities in ageing and Alzheimer’s disease. Neuroimaging studies on structural brain differences showed a larger premorbid brain volume to be an additional protective factor in Alzheimer’s disease (Perneczky *et al.*, 2010; Meng and D'Arcy, 2012; Sumowski *et al.*, 2014). However, protective lifestyle factors and brain volume are gross measures that are not informative about protective functional brain mechanisms. Hence, the overall aim of the current study was to test particular functional brain features that support higher resilience of cognitive performance during the course of Alzheimer’s disease.

The fronto-parietal control network has been proposed to play a key role in mental health and reserve in ageing (Cole *et al.*, 2014; Medaglia *et al.*, 2017). The fronto-parietal control network harbours major brain hubs (Cole *et al.*, 2010) and controls the activity of other functional networks across different cognitive domains (Cole *et al.*, 2013). Thus, this network may be fundamental for maintaining efficient functional brain processes that underlie cognitive performance when brain damage occurs. We recently showed that higher global connectivity of a hub in the fronto-parietal control network is associated with higher reserve in amyloid-positive mild cognitively impaired patients who are at increased risk of developing Alzheimer’s disease dementia (Franzmeier *et al.*, 2017*a*). Specifically, we showed that higher global functional connectivity of the left frontal cortex (gLFC-connectivity, Brodmann area 6/44) was associated with: (i) more years of education (i.e. an epidemiological factor that is associated with lower risk of Alzheimer’s disease); and (ii) an attenuated impact of Alzheimer’s disease-related posterior parietal glucose hypometabolism on memory performance (Franzmeier *et al.*, 2017*b*). Particularly the global functional connectivity of that hub region of the fronto-parietal control network in the left rather than the right hemisphere has been related to reserve-associated protective factors including higher general cognitive ability (i.e. fluid intelligence) and better cognitive control in young to middle-aged subjects (Cole *et al.*, 2012, 2015). Together, these results suggest that gLFC-connectivity may contribute to reserve at an early stage in Alzheimer’s disease, and motivated us to ask whether gLFC-connectivity is associated with higher reserve during the entire course of the disease from preclinical Alzheimer’s disease to dementia.

Here, we hypothesized that at higher levels of gLFC-connectivity the level of impairment in global cognition (Mini-Mental-State Examination, MMSE) and episodic memory (delayed free recall) are shifted relative to levels of biomarkers of Alzheimer’s disease related neurodegeneration. To assess the specificity of our hypothesis we conducted control analyses for global connectivity of additional regions including: (i) the LFCs’ right homotopic region (i.e. right frontal cortex, RFC); and (ii) two null-regions within the visual and motor cortices for which we did not expect any significant effects. We tested our hypothesis based on resting state functional MRI data, cognitive and biomarker levels obtained in two cohorts including: (i) participants with ADAD recruited within the Dominantly Inherited Alzheimer Network (DIAN); and (ii) elderly subjects with sporadic Alzheimer’s disease recruited within the German Center for Neurodegenerative Diseases longitudinal study on cognition and dementia (DELCODE). The study design of DIAN allowed us to examine the association between gLFC-connectivity and cognitive performance against the dynamic changes of biomarkers of Alzheimer’s disease pathology years before onset of dementia. The inclusion of sporadic Alzheimer’s disease provided the opportunity to test whether any effects of gLFC-connectivity on reserve can be generalized towards the more common age-related form of accumulating Alzheimer’s disease pathology in elderly subjects. Thus, the current cross-sectional study assessed for the first time a core candidate functional brain substrate underlying higher cognition during the course of Alzheimer’s disease from preclinical to dementia stages of Alzheimer’s disease.

## Materials and methods

### Participants

#### The DIAN sample of ADAD and controls

Seventy-four mutation carriers exhibiting Alzheimer’s disease causing mutations in genes *PSEN1*, *PSEN2* or *APP*, and 55 non-mutation carrier siblings were included from the DIAN database (Clinical Trial Identifier NCT00869817) (Moulder *et al.*, 2013). Beyond the inclusion criteria of DIAN, the requirement for inclusion in the current study was the presence of baseline resting state functional MRI, T_1_ structural MRI, CSF biomarker assessments, partial volume corrected global Pittsburgh compound B-PET (PiB-PET) binding data, and neuropsychological test scores. Because these stricter inclusion criteria resulted in the selection of a subsample from the larger DIAN cohort (*n* = 177 mutation carriers, and *n* = 136 non-mutation carriers), we tested for selection bias by comparing baseline characteristics [age, gender, years of education, estimated years from symptom onset (EYO)] between the selected sample (*n* = 129) and the excluded (*n* = 184) sample recruited in DIAN, using two-sample *t*-tests for continuous and chi-squared tests for categorical variables. No significant (i.e. *P* > 0.05) differences between groups in any of the variables were found, suggesting that the selected sample is representative of the DIAN cohort. For details on the entire DIAN sample versus the selected DIAN sample please see Supplementary Table 1. Distributions of the main biomarkers and cognitive test scores of the selected DIAN sample can be found in Supplementary Fig. 1. The EYO was defined as the difference between a participant’s age and the parental age of symptom onset, as an estimate of the number of years until symptom onset in mutation carriers. The measurement of global PiB-PET and CSF biomarkers of amyloid-β, tau phosphorylated at serine-181 (p-tau_181_) and total tau have been described previously (Bateman *et al.*, 2012; Benzinger *et al.*, 2013). Local ethical approval and written informed consent had been obtained from each participant by the respective DIAN centres.

#### The DELCODE sample of sporadic Alzheimer’s disease and controls

A total of 116 elderly participants were included from the DELCODE study encompassing participants with cognitively normal status (*n* = 49), subjective cognitive decline (*n* = 40), mild cognitive impairment (MCI, *n* = 14) and Alzheimer’s disease dementia (*n* = 13). DELCODE is an ongoing prospective longitudinal study on the development of neuroimaging and biofluid-derived markers for the early detection of late onset sporadic Alzheimer’s disease. The study is conducted at eight University hospitals within the German Center for Neurodegenerative Diseases. Participants were defined as cognitively normal when showing a Clinical Dementia Rating of zero and neither reporting cognitive complaints nor scoring lower than 1.5 standard deviations (SD) of age-, sex-, and education-adjusted norms on all subtests of the German CERAD-NP Plus battery (Berres *et al.*, 2000), which includes the Trail Making Test versions A and B (Reitan, 1958) and letter fluency in addition to the original version of the CERAD battery (Morris *et al.*, 1989). Subjective cognitive decline was defined by criteria including: (i) Clinical Dementia Rating ≤ 0.5; (ii) normal performance on the tests of CERAD-NP Plus (i.e. a criterion of cognitively normal); and (iii) subjective experience of cognitive decline between the last 6 months and 5 years as reported in semi-structured interviews conducted by a physician with the study participant. Mild cognitive impairment was diagnosed at a Clinical Dementia Rating ≤ 0.5 and cognitive performance level being 1.5 SD below the age- and education adjusted norm for the word-list delayed recall trial of the CERAD-NP-Plus battery (Petersen *et al.*, 2014), thus single- and multi-domain amnestic mild cognitive impairment was included. Alzheimer’s disease dementia was diagnosed when fulfilling the clinical NINDCS/ADRDA criteria of probable Alzheimer’s disease (McKhann *et al.*, 2011). All participants included in the current study underwent lumbar puncture to determine CSF levels of amyloid-β_42_, amyloid-β_40_, p-tau_181,_ total tau and were *a priori* dichotomized as showing either normal or abnormal CSF amyloid-β levels (CSF amyloid-β+ versus CSF amyloid-β−), based on the previously established cut-off for CSF amyloid-β_42/40_ ratio < 0.1 (Janelidze *et al.*, 2016). All participants had to have complete baseline data available including resting state functional MRI, T_1_ structural MRI, CSF biomarker levels, and neurocognitive test scores. When testing for selection bias by comparing baseline characteristics (age, gender, education) between the selected sample (*n* = 116) and the excluded sample (*n* = 250) using two-sample *t*-tests for continuous and chi-squared tests for categorical variables, no significant (i.e. *P* > 0.05) differences between groups in any of the variables were found, suggesting that the selected sample is representative of the whole DELCODE cohort. Details of the entire versus the selected DELCODE sample can be found in Supplementary Table 2. Biomarkers and cognitive test score distributions of the selected DELCODE sample are illustrated in Supplementary Fig. 1.

### Assessment of general cognitive ability and memory performance

As a measure of general cognitive function, we used the MMSE score (Folstein *et al.*, 1975). For episodic memory, the delayed free recall test included in the Wechsler Memory Scale Logical Memory II test was used (Kent, 2013). These two measures of global cognition and episodic memory were selected from a larger set of psychometric tests since those tests were obtained in both DIAN and DELCODE and thus yielded comparability between both studies.

### MRI acquisition

In both DIAN and DELCODE, all MRI scans were acquired on 3 T MRI systems (Siemens) with unified scanning protocols for each study. High resolution structural images were obtained using a T_1_-MPRAGE sequence (DIAN: repetition time/echo time = 2300 ms/2.95 ms, flip angle = 9°, 1.1 × 1.1 × 1.2 mm voxels; DELCODE: repetition time/echo time = 2500 ms/4.33 ms, flip angle = 7°, 1 mm isotropic voxels) with whole-brain coverage. Resting state functional MRI images were recorded using a T_2_*-weighted EPI sequence (DIAN: repetition time/echo time = 2230 ms/30 ms, flip angle = 80°, 3.3 mm isotropic voxels, 140 volumes; DELCODE: repetition time/echo time = 2580 ms/30 ms, flip angle = 80°, 3.5 mm isotropic voxels, 180 volumes). In DELCODE, the participants were instructed to keep their eyes closed and not to fall asleep prior to the resting state scan. In DIAN, resting state functional MRI was acquired with eyes open.

### MRI preprocessing and connectivity analysis

The same processing pipelines were applied to the MRI scans from the DIAN and DELCODE cohort. Note that all processing steps were conducted separately for each sample, i.e. no data were pooled across DELCODE and DIAN but analysed separately within each study.

#### Preprocessing of structural MRI and spatial normalization

T_1_ MRI images were spatially normalized using SPM12 (Wellcome Trust Centre for Neuroimaging, University College London, UK: www.fil.ion.ucl.ac.uk/spm). In a first step, T_1_-weighted images were segmented into probabilistic tissue maps of grey matter, white matter and CSF compartments (Ashburner and Friston, 2005). Non-linear spatial normalization parameters were estimated using SPM’s DARTEL toolbox (Ashburner, 2007), yielding a sample-specific template that was subsequently registered to a template in MNI standard space in order to estimate affine transformation parameters. In a next step, the non-linear DARTEL and affine transformation parameters were jointly applied to the segmented grey matter maps to obtain participant specific grey matter maps in MNI space. All grey matter maps in MNI space were then averaged and binarized at a voxel value >0.3 to obtain a sample-specific grey matter mask for functional connectivity analyses.

As a surrogate for regional brain atrophy that is highly related to Alzheimer’s disease pathology and to memory impairment (Petersen *et al.*, 2000), we assessed the volumes of the bilateral hippocampi using T_1_ MRI data, applying a previously described fully automated approach that was validated using manual segmentation (Mak *et al.*, 2011). For details, see Supplementary material.

#### Preprocessing of resting state functional MRI data

All functional images were visually inspected for artefacts prior to analyses. For each participant, all functional MRI volumes were slice-time corrected, realigned to the first volume, and subsequently registered to the native space high resolution 3D T_1_-weighted image. The non-linear (i.e. DARTEL) and affine registration transformation matrix were again jointly applied to the registered functional MRI volumes to spatially normalize all images to MNI space followed by a smoothing step with an 8 mm full-width at half-maximum Gaussian kernel. To remove noise from the spatially normalized resting state functional MRI data, we removed the linear trend and applied a band-pass filter with a frequency band of 0.01–0.08 Hz. Additionally, we regressed out the six motion parameters and the functional MRI signal averaged across the white matter and CSF. Since functional connectivity analyses can be potentially confounded by motion even after realignment and motion regression (Power *et al.*, 2012), we performed motion scrubbing following a previously established protocol (Power *et al.*, 2014). To this end, we computed the framewise displacement (i.e. the average spatial displacement between two adjacent volumes) for each volume of the resting state scan. Volumes that exhibited a framewise displacement >0.5 mm were censored together with one preceding and two subsequent volumes. Participants for whom >30% of the resting-state volumes had to be censored after scrubbing were not included in the current study.

#### Assessment of global connectivity

**Figure 1:**
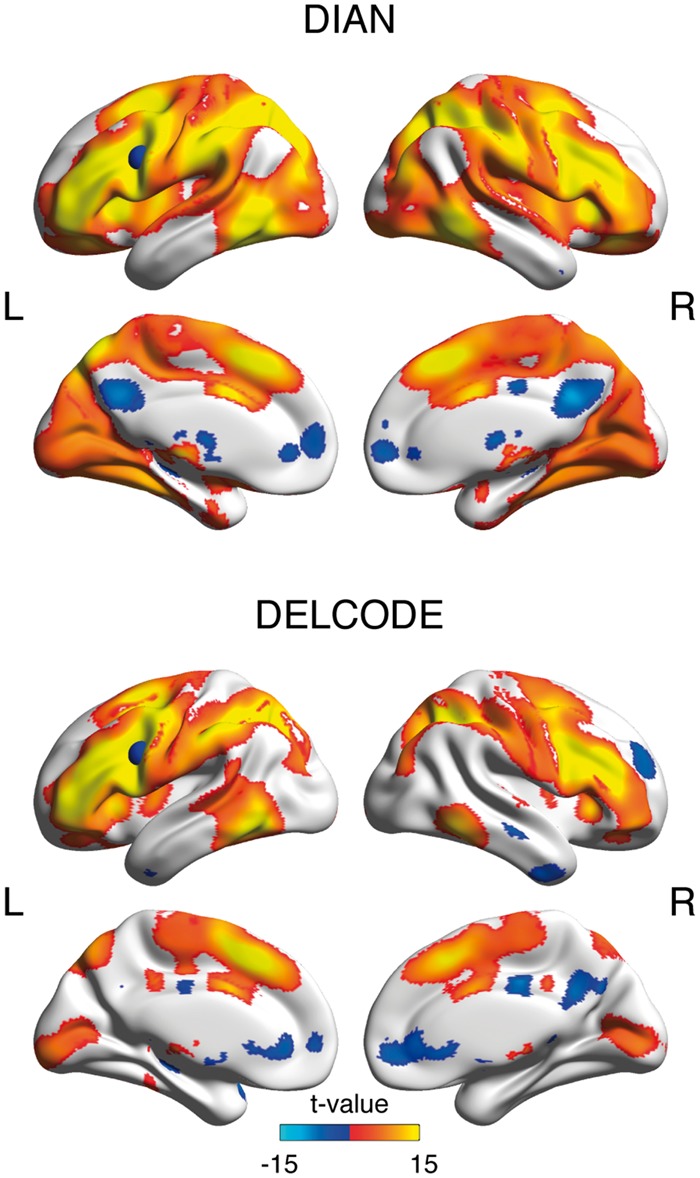
**Seed-based LFC connectivity pattern.** Surface renderings of significant LFC-connectivity in the DIAN and DELCODE sample at a voxel threshold of α < 0.001, family-wise error corrected at the cluster level at α < 0.05. The LFC-region of interest that was used for seed-based functional connectivity analyses is superimposed as a blue sphere on the left hemisphere.

GLFC-connectivity was assessed via seed-based functional connectivity analysis following a previously described approach (Cole *et al.*, 2012; Franzmeier *et al.*, 2017*b*). In brief, we created a binary sphere centred around the left frontal cortex (LFC) (MNI: *x* = −42, *y* = 6, *z* = 28; Brodmann area 6/44; Fig. 1) with a radius of 8 mm, which was used as the seed region for connectivity analyses. The LFC seed region of interest was superimposed onto the preprocessed, scrubbed and grey matter-masked resting state functional MRI data to extract the mean functional MRI time series within the LFC region of interest. Next, we calculated Fisher z-transformed Pearson moment correlations between the LFC-region of interest time series and each remaining grey matter voxel. To obtain the gLFC-connectivity score, we averaged all positive correlations across voxels belonging to the grey matter for each participant consistent with our and others previous analyses of gLFC-connectivity (Cole *et al.*, 2012; Franzmeier *et al.*, 2017*a*, *b*). Note that negative connections were not included, since positive and negative connectivity values would cancel each other out during averaging. In addition to the LFC, global connectivity was also computed for three control regions of interest: (i) the LFCs’ right hemispheric homotopic region (i.e. RFC, *x* = 42, *y* = 6, *z* = 28) to test left hemispheric specificity; and (ii) two null regions including one in the occipital pole (*x* = −19, *y* = −102, *z* = −3), as well as M1 in the motor cortex (*x* = −38, *y* = −22, *z* = 56). Note that we used group-specific rather than subject-specific regions of interest for the current analyses, since there is to date no reliable gold-standard on how to define subject-specific functional clusters that may serve as individual seed-regions of interest.

To address potentially confounding effects of multi-site functional MRI acquisition we included centre as a random effect in our main analyses. In addition, we tested for each study (DIAN and DELCODE) whether global connectivity scores differed between scanner models (Verio, Trio, Prisma in DIAN and Verio, Trio, Skyra in DELCODE) using ANCOVAs controlling for age, gender and education. Here, no significant differences in global connectivity were detected between scanner models for the LFC [DIAN: *F*(4,124) = 0.176, *P* = 0.839; DELCODE *F*(4,111) = 0.674, *P* = 0.570], the RFC [DIAN: *F*(4,124) = 0.144, *P* = 0.867; DELCODE: *F*(4,111) = 0.361, *P* = 0.549], the occipital pole [DIAN: *F*(4,124) = 2.254, *P* = 0.109; DELCODE: *F*(4,111) = 0.1.419, *P* = 0.241] or M1 [DIAN: *F*(4,124) = 0.219, *P* = 0.804; DELCODE: *F*(4,111) = 1.240, *P* = 0.299].

### Statistical analysis

Demographics and cognitive scores were compared between mutation carriers and non-mutation carriers within the DIAN study and between diagnostic groups within the DELCODE study, using ANOVAs (for comparisons between more than two groups), two-sample *t*-tests (for two groups) and chi-square tests (for categorical variables). GLFC-connectivity, CSF-tau, CSF-p-tau_181_ and global partial-volume corrected PIB-PET uptake measures were log- and z-transformed, after which there were no deviations from a normal distribution as assessed via Shapiro-Wilk tests. All subsequent analyses were performed using these transformed values. Note that no data were pooled across the DIAN or DELCODE sample for our analyses.

Next, we tested whether the effect of Alzheimer’s disease severity on cognitive ability is attenuated at higher levels of gLFC-connectivity. For DIAN, the primary linear mixed effects models included the interaction term EYO × gLFC-connectivity and main effects of these variables on either MMSE or logical memory delayed free recall. As a secondary measure of disease stage, we used CSF-tau instead of EYO. All linear mixed effects models were tested separately in mutation carrier and non-mutation carrier groups and were adjusted for gender and family affiliation as fixed effects, and centre as a random effect.

To test whether the gLFC-connectivity × EYO interaction term improves the model fit, we compared the full model to the reduced model that did not include the interaction term as a predictor. We compared the Akaike information criterion between the full and the reduced model to assess whether including the interaction term yielded a lower Akaike information criterion, indicating a better model fit. Consistent with the approach in previous DIAN studies (Bateman *et al.*, 2012), age was not included in the models due to collinearity with EYO. However, DIAN participants are relatively young (mean age = 37 years), thus ageing is unlikely to account for any effects of EYO or CSF-tau on cognition in mutation carriers. These analyses were conducted in an equivalent fashion for global connectivity of the RFC, the occipital pole and M1.

Next, we modelled the interaction effect (EYO × gLFC-connectivity) on global cognition (i.e. MMSE). In addition, we estimated the trajectories of biomarker alterations as done previously based on cross-sectional data (Bateman *et al.*, 2012), so that the course of cognitive alterations could be compared to that of biomarkers of Alzheimer’s disease pathological brain differences. In line with a previous approach (Suarez-Calvet *et al.*, 2016), we fitted polynomial mixed effects models for each outcome variable (MMSE, CSF-tau, precuneus FDG-PET, global PiB-PET binding, and hippocampal volume) as a function of EYO, quadratic (EYO^2^) and cubic (EYO^3^) terms, gender, and family affiliation as fixed effects and site as random effect (Bateman *et al.*, 2012), stratified for mutation carriers and non-mutation carriers. A first order interaction of EYO × gLFC connectivity was included, in addition, when global cognition was the dependent variable. For each outcome variable, the best model was chosen based on the Akaike information criterion. We then plotted the predicted difference between mutation carriers and non-mutation carriers at each EYO, divided by the standard deviation of the respective variable within the pooled sample. Since we adopted the modelling approach from previous studies on biomarker changes in DIAN, the results for the biomarker trajectories were highly consistent with previous reports (Bateman *et al.*, 2012; Benzinger *et al.*, 2013; Suarez-Calvet *et al.*, 2016). This plot was restricted to an EYO range between −20 and +10 because of low numbers of participants at extreme values of EYO and to prevent identifiability of subjects based on extreme values of EYO (as required by DIAN publication guidelines).

Similarly, for DELCODE, we computed linear mixed effects models to test the interaction between CSF-tau (as a marker of disease severity that closely tracks memory decline) (Brier *et al.*, 2016) and gLFC-connectivity on either MMSE or logical memory delayed recall, adjusting for age and gender as fixed effects and for centre as a random effect. These models were computed separately in the control (CSF amyloid-β−) and Alzheimer’s disease (CSF amyloid-β+) groups. In addition, we computed reduced models without the interaction term as a predictor and assessed differences in the model fit (i.e. Akaike information criterion) between the full and the respective reduced model. Separate models were computed for each dependent variable including global connectivity of the RFC, the occipital pole and M1. In an exploratory analysis, we additionally tested the effect of global connectivity × CSF-tau on the word-list delayed recall subscale of the ADAS-Cog battery, which was only available in the DELCODE sample.

To model the effect of gLFC-connectivity on the trajectories of cognitive changes across increasing disease severity (CSF-tau levels), we applied the same polynomial mixed effects approach as used in the DIAN sample, this time stratified by CSF-amyloid-β status. That is, we computed polynomial mixed effects models including CSF-tau (quadratic and cubic terms), controlled for age, gender (fixed effects) and centre (random effect) to predict biomarkers (i.e. CSF-amyloid-β_42/40_ ratio, hippocampal volume). A first order interaction of CSF-tau × gLFC connectivity was included, in addition, when MMSE was the dependent variable.

A schematic illustration of these above described main analyses is illustrated in Supplementary Fig. 2.

In additional models, we tested whether education (as a protective factor associated with higher reserve) predicted gLFC-connectivity in both samples, DIAN and DELCODE. These analyses were adjusted for gender and centre (random effect) as well as EYO and family affiliation in DIAN and age in DELCODE.

All analyses were computed using the freely available *R* statistical software package (http://www.r-project.org) (R Core Team, 2014), standardized beta coefficients were considered significant when meeting an alpha-threshold of α < 0.05. For our main analyses of the interaction gLFC-connectivity times Alzheimer’s disease severity (i.e. EYO or CSF-tau) on global cognition (i.e. MMSE) and episodic memory (i.e. logical memory delayed recall) (four analyses across two samples), we applied a Bonferroni-corrected alpha-threshold of α < 0.0125.

## Results

### Sample characteristics

Baseline characteristics and group differences are displayed in Table 1 for DIAN and Table 2 for DELCODE. Positive functional connectivity of the LFC seed region to other brain regions covered primarily the fronto-parietal control network in both the DIAN and DELCODE samples (Fig. 1), consistent with our previous work (Franzmeier *et al.*, 2017*b*).

**Table 1:** Baseline characteristics of the DIAN sample of ADAD and controls

	ADAD-MC	ADAD-NC	Cohen’s *d*	T-value	*P*-value
	(*n* = 74)	(*n* = 55)			
Age	37.49 (10.05)	37.84 (10.31)	0.034	0.193	0.848
Gender (female/male)	42/32	34/21			0.563
Years of education	14.47 (3.2)	15.51 (2.16)	0.38	2.19	0.030
EYO	−9.82 (11.00)	−9.61 (11.77)	0.02	0.104	0.919
Global PiB-PET	2.12 (1.25)	1.04 (0.05)	1.22	6.430	<0.001
CSF-tau	110 (89.48)	55.19 (22.2)	0.84	4.439	<0.001
CSF-p-tau_181_	60.82 (35.58)	29.77 (9.41)	1.19	6.305	<0.001
gLFC-connectivity	0.27 (0.07)	0.30 (0.07)	0.43	2.445	0.016
Logical memory delayed recall	10.08 (6.13)	13.98 (3.71)	0.77	4.168	<0.001
MMSE	27.04 (5.1)	29.45 (1.02)	0.66	3.455	<0.001

MC = mutation carrier; NC = non-mutation carrier. Values are presented as mean (SD).

**Table 2:** Baseline characteristics of the DELCODE sample of sporadic Alzheimer’s disease

Sporadic AD (Aβ+)	CN	SCD	Cohen’s *d*	MCI	Cohen’s *d*	ADD	Cohen’s *d*	F-value	*P*-value
(*n* = 75)	(*n* = 25)	(*n* = 23)	(SCD versus CN)	(*n* = 14)	(MCI versus CN)	(*n* = 13)	(ADD versus CN)		
Age	67.76 (5.23)^b,c^	72.26 (4.16)^a^	0.95	74.64 (5.34)^a^	1.30	71.31 (6.18)	0.62	6.160	<0.001
Gender (female/male)	16/9	10/13		5/9		9/4			0.164
Years of education	14.64 (2.93)	14.87 (3.81)	0.07	14.71 (3.58)	0.02	14.00 (3.11)	0.21	0.192	0.901
CSF-Aβ_42/40_ ratio	0.08 (0.02)^c,d^	0.08 (0.02)^c,d^	0.00	0.06 (0.02)^a,b^	1.00	0.04 (0.01)^a,b^	2.53	15.456	<0.001
CSF- tau	357.08 (136.91)^c,d^	395.16 (178.58)^d^	0.24	534.97 (172.61)^a,d^	1.14	818.53 (322.62)^a,b,c^	1.86	17.616	<0.001
CSF-p-tau_181_	49.08 (17.06)^d^	53.96 (25.89)^d^	0.22	71.99 (21.37)^d^	1.18	101.6 (45.58)^a,b,c^	1.53	12.220	<0.001
gLFC-connectivity	0.23 (0.05)	0.25 (0.05)	0.40	0.23 (0.05)	0.00	0.22 (0.03)	0.24	1.535	0.213
LM delayed recall	14.57 (8.15)^d^	12.00 (7.18)	0.33	10.60 (6.47)	0.54	6.69 (7.26)^a^	1.02	2.905	0.041
MMSE	29.20 (0.96)^c,d^	29.39 (0.78)^c,d^	0.22	27.71 (1.68)^a,b,d^	1.09	23.85 (2.82)^a,b,c^	2.54	43.066	<0.001

**Controls (Aβ−)**	**CN**	**SCD**	**Cohen’s *d***					**T-value**	***P*-value**
**(*n* = 41)**	**(*n* = 24)**	**(*n* = 17)**	**(SCD versus CN)**						

Age	67.29 (4.6)	71.06 (5.53)	0.74					2.376	0.023
Gender (female/male)	15/9	9/8							0.540
Years of education	14.54 (2.62)	15.35 (3.16)	0.27					0.896	0.392
CSF-Aβ_42/40_ ratio	0.12 (0.02)	0.12 (0.01)	0.00					0.957	0.957
CSF-tau	320.78 (112.02)	355.84 (114.32)	0.31					0.979	0.334
CSF-p-tau_181_	51.57 (16.28)	50.35 (21.1)	0.06					0.591	0.581
gLFC-connectivity	0.25 (0.06)	0.23 (0.04)	0.39					1.067	0.293
LM delayed recall	16.56 (8.26)	9.06 (2.19)	1.24					1.824	0.076
MMSE	29.67 (0.76)	29.06 (0.9)	0.73					2.336	0.024

*Post hoc* Tukey test significant (*P* < 0.05) for ^a^versus cognitively normal (CN); ^b^versus subjective cognitive decline (SCD); ^c^versus mild cognitive impairment (MCI); ^d^versus Alzheimer’s disease dementia (ADD). Values are presented as mean (SD).

Aβ = amyloid-β; LM = logical memory.

### DIAN

**Figure 2:**
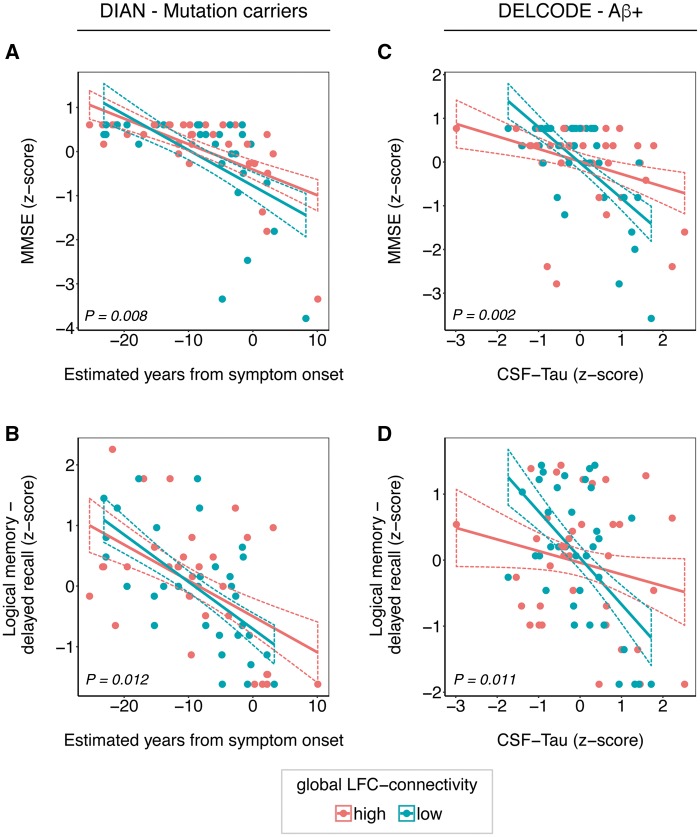
**Interaction gLFC-connectivity × Alzheimer’s disease severity on cognition.** Scatterplots of the interaction gLFC-connectivity × Alzheimer’s disease severity on cognitive performance in the ADAD (DIAN) and sporadic Alzheimer’s disease (DELCODE) sample. For DIAN, the estimated years from symptom onset (EYO) are plotted against the MMSE score (**A**) and the delayed free recall score of the logical memory scale (**B**). For DELCODE, CSF-tau levels are plotted against MMSE (**C**) and logical memory delayed free recall (**D**). For illustrational purposes, groups of high and low gLFC-connectivity (defined via median split) are plotted separately, statistical interactions were calculated using continuous measures. Dashed lines indicate 95% confidence intervals. *P*-values of the interaction terms are displayed for each graph. Aβ = amyloid-β.

To test our major hypothesis that greater gLFC-connectivity moderates the impact of Alzheimer’s disease pathology on cognition in the mutation carrier group of the DIAN sample, we used linear mixed effects models, where we tested the interaction between gLFC-connectivity and EYO on cognitive measures. We found a significant interaction of gLFC-connectivity × EYO on both MMSE [β/standard error (SE) = 0.269/0.099, *P* = 0.008; Fig. 2A] and logical memory delayed free recall (β/SE = 0.275/0.106, *P* = 0.012; Fig. 2B). The full-models including the interaction term showed a better model fit (i.e. Akaike information criterion) as compared to the reduced models for MMSE (186.6 versus 192.1) and logical memory delayed free recall (182.8 versus 186.3). Visual inspection of Fig. 2A and B reveals that at higher levels of gLFC-connectivity, higher EYO (i.e. closer proximity to the estimated age of symptom onset) was associated with relatively less decline in MMSE and logical memory delayed free recall when compared to those with lower levels of gLFC-connectivity within the mutation carrier group. No interaction effect of EYO × gLFC-connectivity on any cognitive measures was found in the non-mutation carrier group. When using CSF-tau instead of EYO as a marker of disease stage (data not shown in Fig. 2), we found a similar result pattern in mutation carriers: at higher levels of gLFC-connectivity, the impact of CSF-tau on cognition was attenuated for MMSE (β/SE = 0.314/0.078, *P* < 0.001). However, such an effect was not found for logical memory delayed free recall in the mutation carrier group. For the non-mutation carrier group, no significant interaction between CSF-tau and gLFC-connectivity on any cognitive measure was observed. All abovementioned analyses were conducted equivalently for the control regions including the RFC, the occipital pole and M1. For the RFC, we found similar but less strong effects as for the LFC, i.e. a significant interaction gRFC-connectivity × EYO on MMSE (β/SE = 0.169/0.078, *P* = 0.018) but no interaction on logical memory delayed free recall (β/SE = 0.189/0.112, *P* = 0.095). These RFC results, however, did not survive Bonferroni correction. The full model including the gRFC-connectivity × EYO interaction term showed a better model fit (i.e. Akaike information criterion) compared to the reduced models for MMSE (172.3 versus 179.1). As expected, no effects were found for the occipital pole or M1. Detailed results of these control analyses are summarized for each control region of interest in Supplementary Tables 3–5.

**Figure 3:**
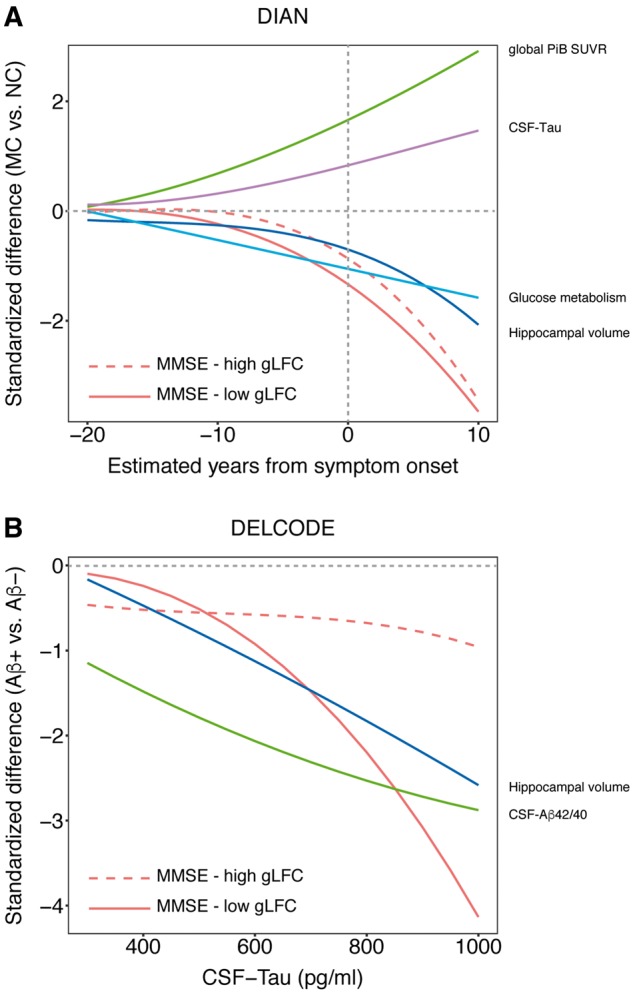
**Cognitive and biomarker changes.** Cognitive and biomarker changes as a function of Alzheimer’s disease severity. MMSE is plotted separately for individuals with high versus low gLFC-connectivity (as defined via median split). For the DIAN sample (**A**) we plotted the standardized difference between mutation carriers (MC) and non-mutation carriers (NC) against the EYO based on the polynomial linear mixed models that best fit each marker. The plot suggests that high gLFC-connectivity is associated with a delay of cognitive decline towards a later timepoint with more progressed levels of Alzheimer’s disease pathology. For the DELCODE sample (**B**) we plotted the standardized difference between CSF-amyloid-β+ and CSF-amyloid-β− subjects against CSF-tau levels. Congruent with the DIAN sample, the plot suggests that cognitive decline is shifted to a later time point in individuals with high levels of gLFC-connectivity.

Figure 3A illustrates the dynamic development of biomarkers and MMSE in mutation carriers versus non-mutation carriers across EYO, as predicted by polynomial mixed models based on cross-sectional data (Bateman *et al.*, 2012). Specifically, the cross-sectionally estimated temporal evolution of impairment in global cognition is shown for mutation carriers with high versus low gLFC-connectivity as defined by median split. The decline in MMSE in individuals with high gLFC-connectivity is shifted to a later time point as compared to individuals with low gLFC-connectivity.

### DELCODE

Next, we aimed to generalize findings on gLFC-connectivity to elderly subjects with evidence for sporadic Alzheimer’s disease pathophysiology (i.e. CSF-amyloid-β+), hence we tested whether greater levels of gLFC-connectivity attenuated the effects of Alzheimer’s disease stage on the same cognitive measures (i.e. MMSE and logical memory delayed recall) in the DELCODE sample. Since EYO is not available in sporadic Alzheimer’s disease subjects, we used CSF total tau levels as a marker of disease severity that is closely linked to neurodegeneration and memory decline (Brier *et al.*, 2016). The interaction gLFC-connectivity × CSF-tau was significant for both global cognition (MMSE: β/SE = 0.285/0.087, *P* = 0.002; Fig. 2C) and logical memory delayed free recall (β/SE = 0.336/0.126, *P* = 0.011; Fig. 2D) within the CSF-amyloid-β+ group. The full models including the interaction term showed a better model fit (i.e. Akaike information criterion) as compared to the null-model for MMSE (194.0 versus 199.2) and logical memory delayed free recall (212.1 versus 215.4). The scatterplots of the interaction effect (Fig. 2C and D) show that the effects of CSF-tau on global cognition (MMSE) and logical memory delayed free recall were attenuated at higher levels of gLFC-connectivity. In an exploratory analysis in the DELCODE cohort, we detected an interaction gLFC-connectivity × CSF-tau on ADAS-Cog word-list delayed recall (β/SE = 0.289/0.137, *P* = 0.039) that was congruent with our main analyses. When tested in the CSF-amyloid-β− control group, all abovementioned interaction models were non-significant. For the RFC, the interaction for gRFC-connectivity × CSF-tau the results were not significant, neither for logical memory delayed free recall (β/SE = 0.215/0.121, *P* = 0.078) nor MMSE (*P* = 0.47). As expected, no effects were detected for the occipital pole or M1. Detailed statistics of these control analyses are displayed for each region of interest in Supplementary Tables 3–5.

**Table 3:** Summary of linear mixed effects models

	β (SE)	T-value	*P*-value	Overall R^2^
**ADAD-MC**				
MMSE^a^				
EYO × gLFC-connectivity	0.269 (0.099)	2.721	0.008^c^	0.498
EYO	−0.576 (0.106)	−5.359	<0.001^c^	
gLFC-connectivity	0.216 (0.094)	2.292	0.025	
LM delayed recall^a^				
EYO × gLFC-connectivity	0.275 (0.106)	2.585	0.012^c^	0.445
EYO	−0.458 (0.109)	−4.208	<0.001^c^	
gLFC-connectivity	0.047 (0.095)	0.494	0.623	
**Sporadic AD−Aβ+**				
MMSE^b^				
CSF-tau × gLFC-connectivity	0.285 (0.087)	3.266	0.002^c^	0.375
CSF-tau	−0.553 (0.071)	−7.758	<0.001^c^	
gLFC-connectivity	0.159 (0.072)	2.207	0.031	
LM delayed recall^b^				
CSF-tau × gLFC-connectivity	0.336 (0.126)	2.672	0.011^c^	0.292
CSF-tau	−0.327 (0.111)	−2.925	0.005^c^	
gLFC-connectivity	−0.011 (0.112)	−0.099	0.922	

^a^Model controlled for gender, family ID (fixed effects) and site (random effect).

^b^Model controlled for age, gender (fixed effects) and site (random effect).

^c^*P* < 0.05, Bonferroni corrected.

Aβ = amyloid-β; AD = Alzheimer’s disease; LM = Logical memory; MC = mutation carrier; NC = non-mutation carrier.

Figure 3B illustrates the change in cognition and biomarkers across the spectrum of CSF-tau levels as predicted by polynomial mixed effects models. With increasing CSF-tau concentrations, there is a decrease in CSF-amyloid-β_42/40_ ratio and hippocampal volume indicating more progressed Alzheimer’s disease. Similar to the results in DIAN, the cross-sectionally estimated trajectory of the decline in MMSE is shifted to a later time point in individuals with high gLFC-connectivity when compared to individuals with low gLFC-connectivity, reflecting the significant interaction gLFC-connectivity × CSF-tau in the CSF-amyloid-β+ group.

### Is global functional connectivity of the left frontal cortex altered in Alzheimer’s disease?

Next, we assessed whether gLFC-connectivity is reduced in subjects with Alzheimer’s disease pathology. For DIAN, an ANCOVA analysis showed that gLFC-connectivity was reduced in mutation carriers compared to non-mutation carriers (*P* = 0.0164), controlled for age, gender, education, and site. However, markers of disease severity including EYO, CSF-tau global PiB-PET uptake and hippocampal volume were not associated with gLFC-connectivity in mutation carriers, as tested by linear mixed effects analyses. These results suggest that gLFC-connectivity is relatively stable across the course of the disease. No associations between gLFC connectivity and any of the variables including EYO, CSF-tau, global PiB-PET uptake, and hippocampal volume were found in the non-mutation carriers. For DELCODE, no differences in gLFC-connectivity were detected between CSF-amyloid-β+ and CSF-amyloid-β- groups and no associations were found between gLFC-connectivity and age or disease severity as assessed by CSF-tau, CSF-amyloid-β_42/40_ ratio or hippocampal volume, neither in the CSF-amyloid-β+ nor CSF-amyloid-β– groups.

### Associations between global functional connectivity of the left frontal cortex and education

We previously reported that education, a protective factor in ageing and neurodegenerative diseases that is often used as a surrogate marker of reserve, is associated with higher gLFC connectivity in prodromal Alzheimer’s disease (Franzmeier *et al.*, 2017*b*, *c*). In general agreement with our previous findings, linear mixed models showed that more years of education were associated with greater gLFC-connectivity in ADAD mutation carriers at trend level significance (β/SE = 0.172/0.117, *P* = 0.072), and in the CSF-amyloid-β+ of the sporadic Alzheimer’s disease sample (β/SE = 0.248/0.114, *P* = 0.033).

## Discussion

The main finding of the current cross-sectional study is that in participants with higher levels of gLFC-connectivity, the effect of Alzheimer’s disease pathology on cognition was attenuated compared to participants with lower levels of gLFC-connectivity. These results were consistent in patients with ADAD (DIAN) and sporadic Alzheimer’s disease (DELCODE), suggesting that higher gLFC-connectivity allows better maintenance of cognitive abilities during the progression of Alzheimer’s disease.

From the perspective of dynamic biomarker development, higher reserve should translate into a shift in the temporal trajectory of cognitive decrease during the progression of Alzheimer’s disease. Modelling the trajectory of cognitive differences in MMSE and delayed recall against increases in Alzheimer’s disease pathology (CSF-tau, EYO) showed for participants with higher gLFC-connectivity a shift of the cross-sectionally estimated trajectories of cognitive decline to the right, i.e. to more progressed levels of Alzheimer’s disease pathology. A strength of the DIAN study in ADAD is the presence of relatively ‘pure’ Alzheimer’s disease pathology that can be tracked well ahead of dementia onset. Still, the generalizability of findings to the more common sporadic form of Alzheimer’s disease is important. Using CSF-tau as a marker of disease severity that is closely linked to cognitive symptoms in CSF-amyloid-β+ subjects of the DELCODE study, we replicated the findings of a higher preservation of episodic memory and global cognition at more progressed levels of Alzheimer’s disease in participants with higher levels of gLFC-connectivity. This result is also consistent with our previous study in prodromal Alzheimer’s disease, where higher gLFC-connectivity was associated with attenuated impact of Alzheimer’s disease-related parietal glucose hypometabolism on memory (Franzmeier *et al.*, 2017*b*). Thus, the detection of the association between gLFC-connectivity and reserve is not specific to ADAD.

Since in ADAD the pathological development shows a genetically-driven and stereotypical progression of Alzheimer’s disease pathology, the question of why some participants show higher or lower cognitive performance than expected based on the pathological level is particularly intriguing (Aguirre-Acevedo *et al.*, 2016). In fact, the variability in cognitive decreases at any level of neurodegeneration in ageing and Alzheimer’s disease is not a mere random error of prediction based on pathology, but shows a systematic bias (Reed *et al.*, 2010; Zahodne *et al.*, 2013, 2015). Demographic factors such as education or premorbid verbal cognitive abilities are associated with higher than expected memory performance in ageing and Alzheimer’s disease (Rentz *et al.*, 2010; Ewers *et al.*, 2013; Soldan *et al.*, 2013; Topiwala *et al.*, 2015). However, such protective factors are global in nature and difficult to attribute to structural or functional brain changes (Solé-Padullés *et al.*, 2009; Arenaza-Urquijo *et al.*, 2013; Barulli and Stern, 2013). The current findings thus address the need for an identification of functional brain differences that underlie reserve, i.e. the ability to maintain cognition at a higher level in the presence of pathology.

Why is global connectivity of the LFC hub an important characteristic of brain function and, in particular, reserve? Global connectivity is a graph theoretical measure of the class centrality that captures the connectivity of a node in the whole connectome (Bullmore and Sporns, 2009). The LFC is among the top 5% of globally connected brain regions (Cole *et al.*, 2010). Changes in hubs have particularly strong global impact on the brain compared to connectivity differences in other brain regions and are critical for the resilience of brain networks to targeted attack (Achard *et al.*, 2006; Alstott *et al.*, 2009; Santarnecchi *et al.*, 2015). Reduction of hub connectivity has been associated with different neurodegenerative and psychiatric diseases (Buckner *et al.*, 2009; Crossley *et al.*, 2014), while increased hub connectivity is associated with better cognition (Cole *et al.*, 2012; Liu *et al.*, 2017). These results support the notion that connectivity of hubs is important for sustaining cognition when developing Alzheimer’s disease.

The LFC is a hub closely linked to the fronto-parietal control network. This functional network is particularly important in flexibly regulating the activity of different functional networks (Chen *et al.*, 2013). Such a network regulation by the fronto-parietal control network is critical for successful cognitive task performance (Kragel and Polyn, 2015), particularly when other functional networks become impaired (Cole *et al.*, 2014). Thus, as a hub of the fronto-parietal control network, the LFC may be of critical importance for the regulation of other networks to support successful cognition (Cole *et al.*, 2015).

As shown by our control analyses, we could detect only a single significant interaction effect of RFC × EYO on MMSE, which did not survive Bonferroni correction. No effects for unimodal brain regions including M1 and the visual cortex were observed. This result pattern suggests that the findings are specific to the LFC, where predominantly the LFC rather than the RFC supports reserve. Our findings echo previous reports of resting state functional MRI assessed connectivity in a similar LFC area to be critical for supporting functional network resilience during simulated targeted attack of hub nodes (Santarnecchi *et al.*, 2015). Together, these findings suggest that gLFC-connectivity is a feasible substrate of higher resilience of cognition in the face of neuropathology.

We found no decreases of gLFC-connectivity in sporadic Alzheimer’s disease and only a mild reduction in ADAD, suggesting the gLFC-connectivity is relatively stable in Alzheimer’s disease. Previous studies reported that brain hubs are in general implicated in neurodegenerative and psychiatric diseases (Crossley *et al.*, 2014); however, the posterior and hippocampal hubs are selectivity impaired in Alzheimer’s disease (Yu *et al.*, 2017), potentially due to their overlap with sites of early Alzheimer’s disease pathology (Buckner *et al.*, 2009). This has been consistently shown in sporadic and autosomal dominantly inherited Alzheimer’s disease, where key hubs such as the posterior cingulate, precuneus or hippocampus show disruptions early in the disease course even prior to the onset of cognitive symptoms (Sorg *et al.*, 2007; Mevel *et al.*, 2011; Damoiseaux *et al.*, 2012; Chhatwal *et al.*, 2013). In contrast, we could show that connectivity of the LFC hub region remains relatively unchanged across different Alzheimer’s disease severity levels. Thus, the relatively spared LFC hub may be well posed to subserve the maintenance of cognition during the course of Alzheimer’s disease. At the same time, gLFC-connectivity showed at no stage of Alzheimer’s disease an increase, thus we found no indication of a temporary compensatory increase in response to pathology. Rather, differences in gLFC-connectivity may have existed before disease onset. In line with our previous study (Franzmeier *et al.*, 2017*c*), we observed in the current study an association between more years of education and higher gLFC-connectivity, suggesting that gLFC-connectivity is possibly shaped by early life experiences, consistent with view of cognitive reserve (Stern, 2012). Thus, higher gLFC-connectivity is most likely a pre-morbid existing trait that enables to better cope with pathological changes during diseases such as Alzheimer’s disease.

For the interpretation of the current results, potential caveats should be taken into consideration. First, as a measure of hubness, we used global connectivity, which in graph theoretical terms is also called weighted degree centrality. It should be noted that alternative measures of centrality have been proposed such as the page-rank centrality and eigenvector centrality (Zuo *et al.*, 2012). However, we chose the current global connectivity measure as it is the most widely used index of centrality applied in human brain connectomics (Buckner *et al.*, 2009; Wang *et al.*, 2010; Cole *et al.*, 2012, 2015; Zuo *et al.*, 2012; Santarnecchi *et al.*, 2015; Franzmeier *et al.*, 2017*a*, *b*). Thus, in order to facilitate comparability with previous findings, we focused on the current measure of global connectivity.

Second, the current study included mostly subjects within the range of asymptomatic or mild cognitive impairment of Alzheimer’s disease. Thus, a generalization to more severe dementia stages needs to be tested in future studies. It is conceivable that the influence of the LFC or any substrate of reserve wanes as the disease becomes more severe and network failure becomes more profound (Jones *et al.*, 2016).

Third, in order to facilitate comparability across samples we focused our analyses on the MMSE and logical memory delayed free recall scores. A more comprehensive testing of episodic memory is desirable, but was not possible since the overlap of memory tests between the studies was limited. In DELCODE, the widely used verbal word list learning test as included in the ADAS-Cog battery was also available, for which we found the same result pattern as for the logical memory delayed free recall and MMSE. The current results are also consistent with our previous results on gLFC-connectivity on memory, where more comprehensive memory tests were used (Franzmeier *et al.*, 2017*b*). Still, future studies may utilize a wider breadth of neuropsychological tests to capture global cognitive and memory abilities in a more comprehensive manner.

Fourth, we included only cross-sectional data rather than longitudinal assessment, thus, individual trajectories of cognitive changes could not be assessed. However, the trajectories assessed across the spectrum of Alzheimer’s disease severity at the cross-sectional level are highly consistent with the longitudinal trajectories of biomarker changes. Thus, especially in ADAD, the cross-sectionally estimated trajectories may be a proxy of the expected trajectories when assessed longitudinally, which awaits further validation once sufficient longitudinal data become available in studies such as DIAN or DELCODE.

Lastly, we assessed only Alzheimer’s disease among other neurodegenerative diseases. Resilience of cognition to pathologies has also been reported in fronto-temporal dementia, vascular dementia or multiple sclerosis (Zieren *et al.*, 2013; Martins Da Silva *et al.*, 2015; Premi *et al.*, 2017). It is an open question whether gLFC-connectivity subserves reserve also in those disorders.

As an outlook, the current identification of the LFC hub may provide a suitable target for therapeutic intervention. Previous studies have shown that connectivity of frontal hubs can be enhanced by cognitive training (Takeuchi *et al.*, 2017) and higher frontal connectivity can be induced by transcranial magnetic stimulation or transcranial direct-current stimulation (Chen *et al.*, 2013; Kim *et al.*, 2016)*.* Enhancement of gLFC-connectivity by stimulation techniques, cognitive training, or physical exercise (Duzel *et al.*, 2016) may provide an attractive non-invasive secondary prevention approach to sustain cognitive resilience in Alzheimer’s disease (Clark and Parasuraman, 2014).

## Supplementary Material

Supplementary Tables

Supplementary Figure S1

Supplementary Figure S2

Supplementary Methods

## Abbreviations

ADAD: autosomal dominantly inherited Alzheimer’s disease
DELCODE: German Center for Neurodegenerative Diseases longitudinal study on cognition and dementia
DIAN: Dominantly Inherited Alzheimer Network
EYO: estimated years from symptom onset
gLFC-connectivity: global functional connectivity of the left frontal cortex
LFC: left frontal cortex
MMSE: Mini-Mental-State Examination
RFC: right frontal cortex
