# Supplementary material for: Left frontal hub connectivity delays cognitive impairment in autosomal-dominant and sporadic Alzheimer’s disease
Source: Brain. 2018 Feb 15;141(4):1186–200. doi: 10.1093/brain/awy008 (PMC5888938; doi:10.1093/brain/awy008)
Supplement: Supplementary Tables [file brain-2017-01352-file006_awy008.pdf]

## Supplementary table 1:

| DIAN | Whole sample (n=313) | Non-selected sample (n=184) | Selected sample (n=129) | Selected vs. non-selected p-value |
|---|---|---|---|---|
| Age | 38.73 (10.46) | 39.5 (10.64) | 37.64 (10.13) | 0.119 |
| Gender (m/f) | 190/123 | 70/114 | 53/76 | 0.588 |
| Years of education | 14.32 (3.07) | 13.91 (3.16) | 14.91 (2.84) | 0.064 |
| Estimated years from symptom onset | -8.80 (11.09) | -7.87 (10.85) | -9.73 (11.29) | 0.176 |

## Supplementary table 2:

| DELCODE | Whole sample (n=366) | Non-selected sample (n=250) | Selected Sample (n=116) | Selected vs. non-selected p-value |
|---|---|---|---|---|
| Age | 70.44 (5.91) | 70.52 (6.06) | 70.27 (5.59) | 0.696 |
| Gender (m/f) | 175/191 | 123/127 | 52/64 | 0.436 |
| Years of education | 14.45 (3.04) | 14.34 (2.98) | 14.71 (3.15) | 0.288 |

***Supplementary table 3: Summary of linear mixed effects models for global RFC-connectivity***

| **ADAD-MC** | **β (SE)** | **T-value** | **p-value** | **Overall $R^2$** |
|---|---|---|---|---|
| ***MMSE***[a] | | | | |
| *EYO x gRFC-connectivity* | 0.169 (0.070) | 2.425 | 0.0181 | 0.475 |
| *EYO* | -0.349 (0.071) | -4.954 | <0.001[c] | |
| *gRFC-connectivity* | 0.103 (0.063) | 1.644 | 0.105 | |
| ***LM delayed recall***[a] | | | | |
| *EYO x gRFC-connectivity* | 0.189 (0.112) | 1.695 | 0.095 | 0.457 |
| *EYO* | -0.409 (0.111) | -3.676 | <0.001[c] | |
| *gRFC-connectivity* | 0.111 (0.097) | 1.139 | 0.259 | |
| **Sporadic AD-Aβ+** | **β (SE)** | **T-value** | **p-value** | **Overall $R^2$** |
| ***MMSE***[b] | | | | |
| *CSF-Tau x gRFC-connectivity* | -0.080 (0.109) | -0.733 | 0.466 | 0.247 |
| *CSF-Tau* | -0.471 (0.105) | -4.501 | <0.001[c] | |
| *gRFC-connectivity* | -0.027 (0.113) | -0.242 | 0.810 | |
| ***LM delayed recall***[b] | | | | |
| *CSF-Tau x gRFC-connectivity* | 0.215 (0.121) | 1.787 | 0.078 | 0.256 |
| *CSF-Tau* | -0.259 (0.113) | -2.296 | 0.025 | |
| *gRFC-connectivity* | 0.217 (0.121) | 1.788 | 0.078 | |

*a=model controlled for gender, family ID (fixed effects) & site (random effect); b= model controlled for age, gender (fixed effects) & site (random effect); c=p<0.05, bonferroni corrected*

***Supplementary table 4: Summary of linear mixed effects models for global occipital pole-connectivity***

| **ADAD-MC** | **β (SE)** | **T-value** | **p-value** | **Overall $R^2$** |
|---|---|---|---|---|
| ***MMSE*** [a] | | | | |
| *EYO x gOP-connectivity* | 0.061 (0.116) | 0.523 | 0.602 | 0.436 |
| *EYO* | -0.655 (0.107) | -6.150 | <0.001[c] | |
| *gOP-connectivity* | 0.138 (0.095) | 1.449 | 0.152 | |
| ***LM delayed recall*** [a] | | | | |
| *EYO x gOP-connectivity* | 0.030 (0.115) | 0.264 | 0.792 | 0.446 |
| *EYO* | -0.472 (0.115) | -4.104 | <0.001[c] | |
| *gOP-connectivity* | 0.026 (0.099) | 0.261 | 0.795 | |

| **Sporadic AD-Aβ+** | **β (SE)** | **T-value** | **p-value** | **Overall $R^2$** |
|---|---|---|---|---|
| ***MMSE*** [b] | | | | |
| *CSF-Tau x gOP-connectivity* | -0.109 (0.115) | -0.951 | 0.345 | 0.249 |
| *CSF-Tau* | -0.276 (0.117) | -2.367 | 0.021 | |
| *gOP-connectivity* | 0.188 (0.116) | 1.624 | 0.109 | |
| ***LM delayed recall*** [b] | | | | |
| *CSF-Tau x gOP-connectivity* | -0.043 (0.108) | -0.399 | 0.691 | 0.240 |
| *CSF-Tau* | -0.247 (0.117) | -2.120 | 0.038 | |
| *gOP-connectivity* | 0.203 (0.117) | 1.240 | 0.234 | |

*OP=occipital pole; a=model controlled for gender, family ID (fixed effects) & site (random effect); b= model controlled for age, gender (fixed effects) & site (random effect); c=p<0.05, bonferroni corrected*

## *Supplementary table 5: Summary of linear mixed effects models for global M1-connectivity*

| **ADAD-MC** | **β (SE)** | **T-value** | **p-value** | **Overall $R^2$** |
|---|---|---|---|---|
| ***MMSE[a]*** | | | | |
| *EYO x gM1-connectivity* | 0.078 (0.099) | 0.791 | 0.432 | 0.473 |
| *EYO* | -0.514 (0.110) | -4.664 | <0.001[c] | |
| *gM1-connectivity* | 0.056 (0.101) | 0.551 | 0.583 | |
| ***LM delayed recall[a]*** | | | | |
| *EYO x g M1-connectivity* | 0.069 (0.102) | 0.683 | 0.497 | 0.444 |
| *EYO* | -0.463 (0.113) | -4.091 | <0.001[c] | |
| *gM1-connectivity* | -0.010 (0.104) | -0.094 | 0.925 | |
| **Sporadic AD-Aβ+** | **β (SE)** | **T-value** | **p-value** | **Overall $R^2$** |
| ***MMSE[b]*** | | | | |
| *CSF-Tau x gM1-connectivity* | -0.044 (0.112) | -0.395 | 0.693 | 0.213 |
| *CSF-Tau* | -0.292 (0.119) | -2.465 | 0.016 | |
| *gM1-connectivity* | 0.118 (0.122) | 0.967 | 0.337 | |
| ***LM delayed recall[b]*** | | | | |
| *CSF-Tau x gM1-connectivity* | -0.031 (0.118) | -0.265 | 0.792 | 0.215 |
| *CSF-Tau* | -0.283 (0.114) | -2.478 | 0.016 | |
| *gM1-connectivity* | 0.116 (0.122 | 0.946 | 0.348 | |

*a=model controlled for gender, family ID (fixed effects) & site (random effect); b= model controlled for age, gender (fixed effects) & site (random effect); c=p<0.05, bonferroni corrected*
