# Supplementary figures and images for: Left frontal hub connectivity delays cognitive impairment in autosomal-dominant and sporadic Alzheimer’s disease
Source: Brain. 2018 Feb 15;141(4):1186–200. doi: 10.1093/brain/awy008 (PMC5888938; doi:10.1093/brain/awy008)

## Controls

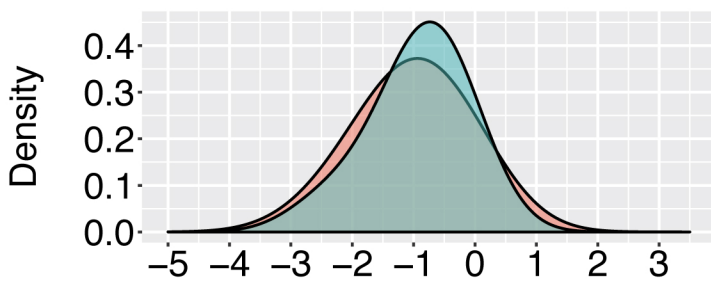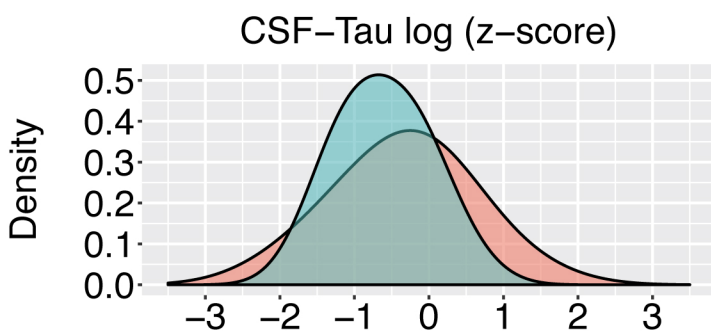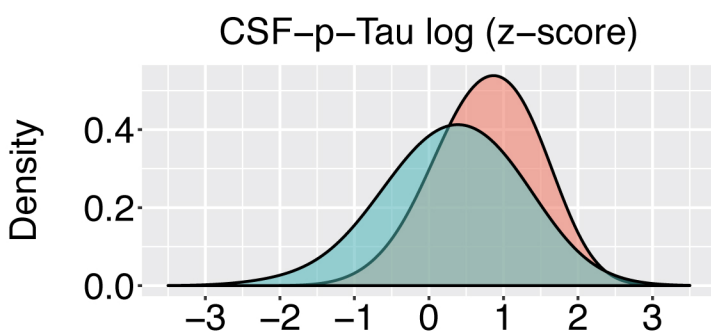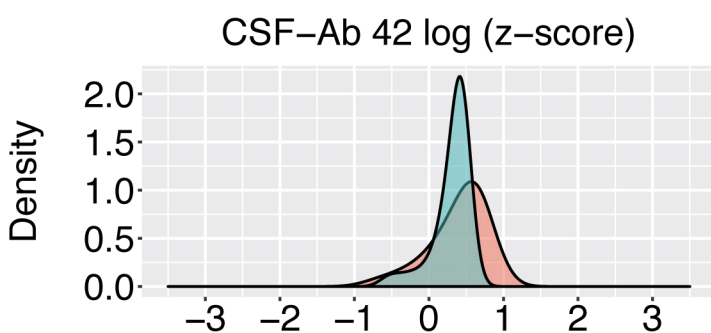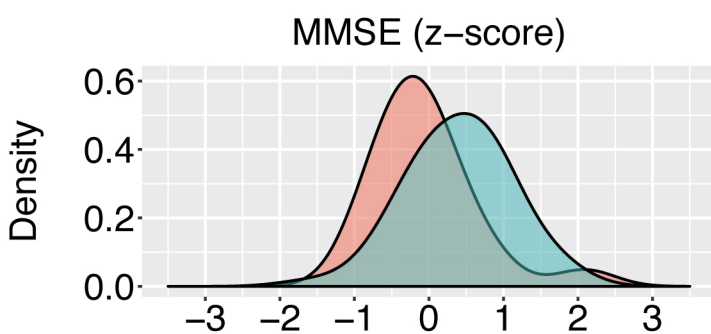

DELCODE Aβ-  
DIAN Non-carriers

## Alzheimer's disease

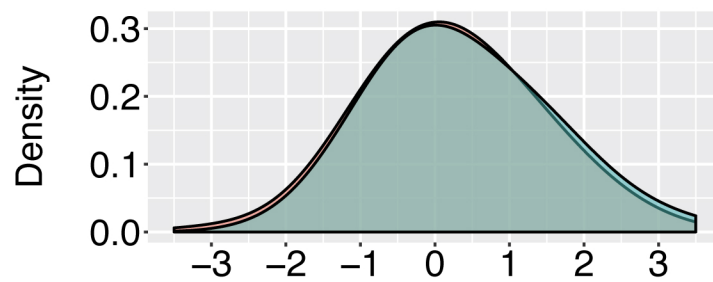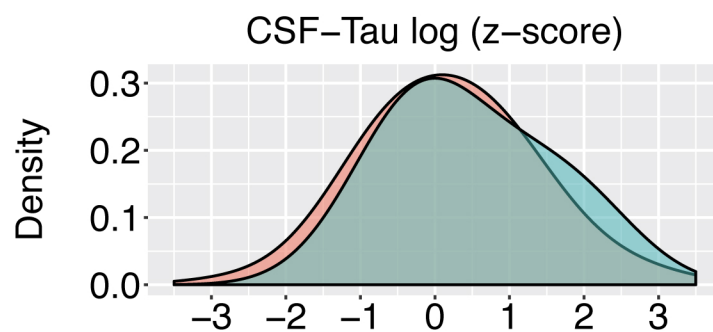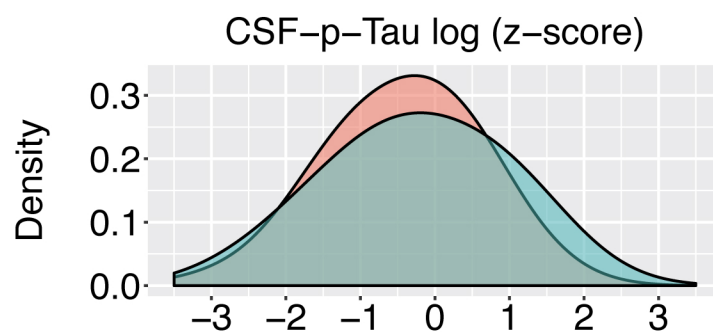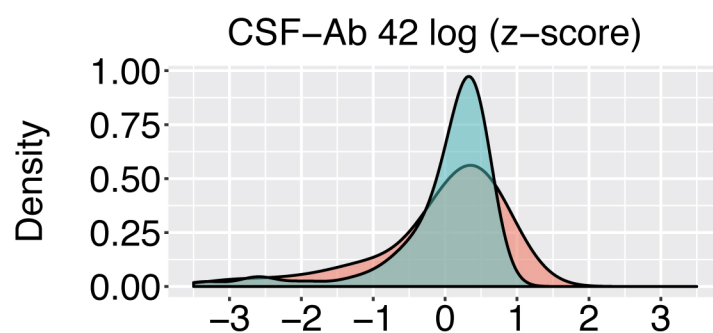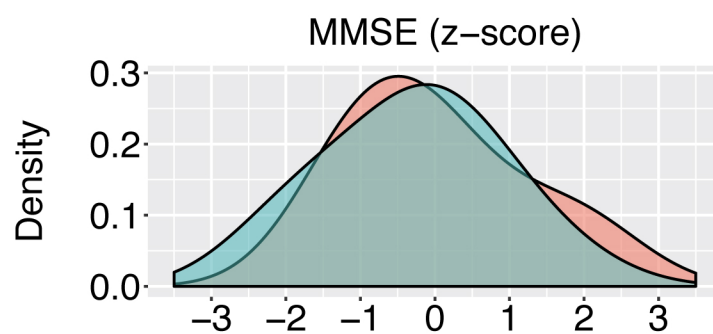

DELCODE Aβ+  
DIAN Mutation-carriers

Supplement: Supplementary Figure S1 [file brain-2017-01352-file007_awy008.pdf]
