# Supplementary material for: Left frontal hub connectivity delays cognitive impairment in autosomal-dominant and sporadic Alzheimer’s disease
Source: Brain. 2018 Feb 15;141(4):1186–200. doi: 10.1093/brain/awy008 (PMC5888938; doi:10.1093/brain/awy008)
Supplement: Supplementary Figure S2 [file brain-2017-01352-file008_awy008.pdf]

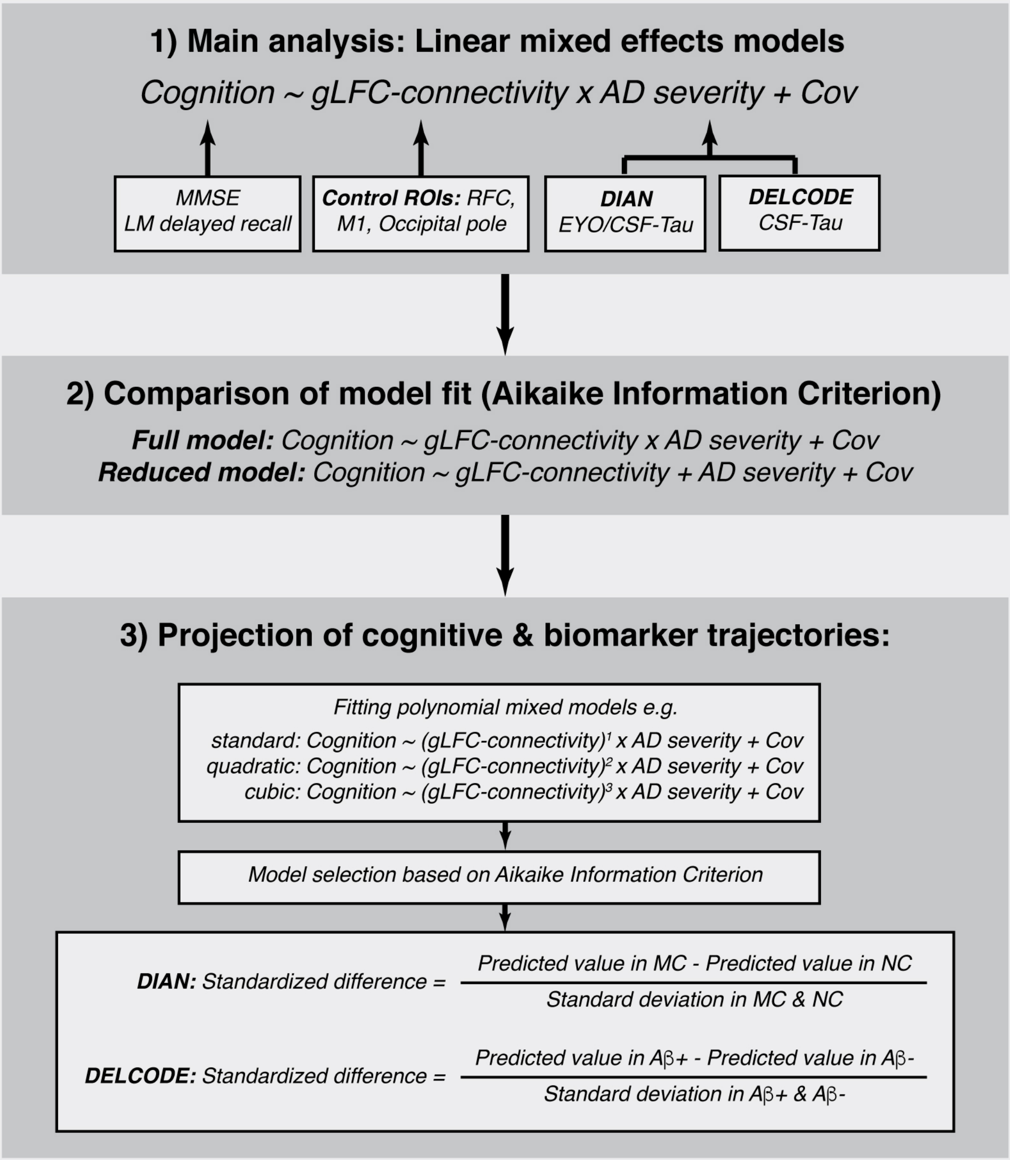
1) Main analysis: Linear mixed effects models
Cognition ~ gLFC-connectivity x AD severity + Cov
MMSE
LM delayed recall
Control ROIs: RFC, M1, Occipital pole
DIAN
EYO/CSF-Tau
DELCODE
CSF-Tau
2) Comparison of model fit (Aikaike Information Criterion)
Full model: Cognition ~ gLFC-connectivity x AD severity + Cov
Reduced model: Cognition ~ gLFC-connectivity + AD severity + Cov
3) Projection of cognitive & biomarker trajectories:
Fitting polynomial mixed models e.g.
standard: Cognition ~ (gLFC-connectivity)¹ x AD severity + Cov
quadratic: Cognition ~ (gLFC-connectivity)² x AD severity + Cov
cubic: Cognition ~ (gLFC-connectivity)³ x AD severity + Cov
Model selection based on Aikaike Information Criterion
DIAN: Standardized difference = Predicted value in MC - Predicted value in NC / Standard deviation in MC & NC
DELCODE: Standardized difference = Predicted value in Aβ+ - Predicted value in Aβ- / Standard deviation in Aβ+ & Aβ-
