# Supplementary material for: Left frontal hub connectivity delays cognitive impairment in autosomal-dominant and sporadic Alzheimer’s disease
Source: Brain. 2018 Feb 15;141(4):1186–200. doi: 10.1093/brain/awy008 (PMC5888938; doi:10.1093/brain/awy008)
Supplement: Supplementary Methods [file brain-2017-01352-file005_awy008.pdf]

Supplementary methods:

<u>*Assessment of hippocampal volume*</u>

As a surrogate for regional brain atrophy that is highly related to AD pathology and to memory impairment (Petersen *et al.*, 2000), we assessed the volumes of the bilateral hippocampi, using a previously described fully automated approach that was previously validated using manual segmentation (Mak *et al.*, 2011). Briefly, we applied the DARTEL flow-fields that were estimated during the spatial normalization step (see above) to normalize each participant's grey matter map to MNI space. During normalization the images were modulated using the Jacobian determinants to preserve local grey matter concentrations (Good *et al.*, 2001). Each participants' normalized and modulated grey matter map was subsequently masked with a bilateral hippocampus mask selected from the widely used Automatic Anatomic Labeling atlas (Tzourio-Mazoyer *et al.*, 2002). From these masked images, we then extracted the bilateral hippocampal volume (Jack *et al.*, 2000; Petersen *et al.*, 2000).

References:

Good CD, Johnsrude IS, Ashburner J, Henson RN, Friston KJ, Frackowiak RS. A voxel-based morphometric study of ageing in 465 normal adult human brains. NeuroImage 2001; 14(1 Pt 1): 21-36.

Jack CR, Jr., Petersen RC, Xu Y, O'Brien PC, Smith GE, Ivnik RJ, *et al.* Rates of hippocampal atrophy correlate with change in clinical status in aging and AD. Neurology 2000; 55(4): 484-89.

Mak HK, Zhang Z, Yau KK, Zhang L, Chan Q, Chu LW. Efficacy of voxel-based morphometry with DARTEL and standard registration as imaging biomarkers in Alzheimer's disease patients and cognitively normal older adults at 3.0 Tesla MR imaging. Journal of Alzheimer's disease : JAD 2011; 23(4): 655-64.

Petersen RC, Jack CR, Jr., Xu YC, Waring SC, O'Brien PC, Smith GE, *et al.* Memory and MRI-based hippocampal volumes in aging and AD. Neurology 2000; 54(3): 581-7.

Tzourio-Mazoyer N, Landeau B, Papathanassiou D, Crivello F, Etard O, Delcroix N, *et al.* Automated anatomical labeling of activations in SPM using a macroscopic anatomical parcellation of the MNI MRI single-subject brain. NeuroImage 2002; 15(1): 273-89.
